# Basic morphometry, microcomputed tomography and mechanical evaluation of the tibiotarsal bone of a dual-purpose and a broiler chicken line

**DOI:** 10.1371/journal.pone.0230070

**Published:** 2020-03-11

**Authors:** George Harash, Kenneth C. Richardson, Zaher Alshamy, Hana Hünigen, Hafez Mohamed Hafez, Johanna Plendl, Salah Al Masri

**Affiliations:** 1 Department of Veterinary Medicine, Institute of Veterinary Anatomy, Freie Universität Berlin, Berlin, Germany; 2 College of Veterinary Medicine, School of Veterinary and Life Sciences, Murdoch University, Murdoch, Australia; 3 Department of Veterinary Medicine, Institute of Poultry Diseases, Freie Universität Berlin, Berlin, Germany; University of California Davis, UNITED STATES

## Abstract

Continuous loading of the skeleton by the body’s weight is an important factor in establishing and maintaining bone morphology, architecture and strength. However, in fast-growing chickens the appendicular skeleton growth is suboptimal making these chickens predisposed to skeletal mineralization disorders and fractures. This study compared the macro- and microstructure as well as the mechanical properties of the tibiotarsus of a novel dual-purpose, Lohmann Dual (LD) and a highly developed broiler, Ross (Ross 308) chicken line. Eighty one-day-old male chicks of each line were grown until their body weight (BW) reached 2000g. Starting at the day of hatching, six birds of each line were sampled weekly. The weight, length and width of the tibiotarsus were measured and its mechanical properties (rigidity, M-Max and the M-fracture) were evaluated using the three-point bending test. Additionally, the mineral density of both, trabecular and cortical bone, the bone volume fraction, the trabecular number, thickness and separation plus cortical thickness of both chicken lines were analyzed using microcomputed tomography. The growth of the tibiotarsus in both chicken lines followed a similar pattern. At the same age, the lighter LD chickens had shorter, thinner and lighter tibiotarsi than those of Ross chickens. However, the LD chickens had a similar cortical thickness, bone volume fraction and similar mineral density of both trabecular and cortical bone to that of Ross chickens. Furthermore, the tibiotarsus of LD chickens was longer, heavier and wider than those of Ross chickens of the same BW. In addition the rigidity of the LD tibiotarsus was greater than that of Ross chickens. This suggests that the tibiotarsus of LD chickens had more bending resistance than those of Ross chickens of the same BW. Consequently, fattening LD chickens to the marketable weight should not affect their leg skeleton stability.

## Introduction

Worldwide, male chickens from layer genetic lines are killed immediately after hatching due to their inability to lay eggs and their slow muscle growth [1]. Farming dual-purpose chickens is an alternative to the culling of males. Here females are used to produce eggs and the males are grown for meat production. This approach is exemplified by the new commercial dual-purpose chicken, the Lohmann Dual (LD), that has been developed by crossing meat and layer chicken lines [2]. Earlier comparative studies show that LD chickens have a better myocardial capillary supply and better aortic mechanical properties compared to the highly specialized broiler, Ross 308, chickens [3]. Nevertheless, LD chickens have a slower growth rate compared to Ross 308 chickens that appears to be associated with the LD chickens having a smaller intestinal absorptive surface area [4].

A recent comparative study between two different slow-growing broiler genotypes showed that the tibiotarsus traits influenced by genetic strain [5]. In earlier studies on rapidly growing meat-producing poultry the tibiotarsal bone was found to be the most affected bone in clinical and subclinical leg problems as tibial dyschondroplasia and rickets [6–8]. Additionally, the tibiotarsus was shown to have significantly higher mechanical and geometrical parameter values as well as higher mineralization than did other bones of the pelvic limb [9, 10]. Consequently, the tibiotarsal bone has greater mechanical resistance to deformation and as such is the most appropriate of the leg bones for research. Although the LD chicken line is grown commercially there is little information on their principal weight bearing bones notably the long bones of the pelvic limb. Therefore, studies on the ultrastructure and bending properties of the LD tibiotarsus are important so that production protocols can be adopted to lessen potential skeletal disorders and thus ensure that their welfare is optimised.

As postulated in Wolff’s Law, during development and aging, bone architecture adapts to withstand the extremes of functional load-bearing [11, 12]. However, this adaptive process appears to be suboptimal in chickens that have been selected for rapid and high growth rates [13]. Thus in rapidly growing broiler lines the tibiotarsal bones are strongly loaded by the body weight of the birds and are more prone to mineralization disorders and fractures [14]. Moreover, market age chickens often suffer from lameness and bone deformities that can cause bone fracture during capture and transportation [15]. A probable reason for the poor pelvic limb bone health in broiler chickens is a reduction in bone quality. The tibiotarsus in broiler chickens is less mineralized and less dense than found in slow-growing genetic lines [13]. Several studies have shown that an increased load on the bone increases bone mass [16, 17].

Previous studies assert that the bone quality of slow-growing broilers is better compared to that of the fast-growing ones and that the faster growing chickens are disadvantaged by their heavier body weight, i.e. the cortical bone of fast-growing broilers is highly porous and poorly mineralized. They report that the association between tibiotarsal fracture strength and growth rate was negative [13, 18–21].

An evaluation of the quality and integrity of the tibiotarsus includes the examination of morphological variables such as bone mass and length [22] as well as details of the microstructure properties of both trabecular and cortical bones including bone mineral density, plus the assessment of mechanical properties such as bone fracture strength and stiffness [20, 21, 23, 24]. Tibiotarsal bone strength is influenced by numerous properties including shape, size, mass, structure, and composition [25–27]. Furthermore, bone strength is correlated positively with trabecular properties [trabecular number, thickness and separation] [28, 29], bone mineral density and bone weight [30, 31].

The aim of this study was to investigate the macro- and microstructural as well as the mechanical properties of the tibiotarsal bone of a dual-purpose chicken line (Lohmann Dual, LD) and a modern broiler chicken line (Ross 308) throughout the period from time of hatching until they reach their market body weight.

## Material and methods

### Animals and husbandry

The tibiotarsal bones of male chicks from two different lines, a dual-purpose line (Lohmann Dual, LD) and a broiler line (Ross 308) were used for this study. The same chickens had previously been used to investigate the gastrointestinal tract [4] as well as the heart and aorta [3]. Animals and husbandry conditions were described in detail in the above studies [3, 4]. Briefly, the chickens were reared under similar husbandry conditions until they reached a body weight (BW) of 2000 g, i. e. 35 days for Ross chickens and 63 days for LD chickens. The study was approved by the Animal Welfare Committee “Landesamt für Gesundheit und Soziales”, Berlin, Germany, ID: 0236/15.

### Sample collection

Six birds from each line were sampled randomly on days 1, 7, 14, 19, 21, 25, 28, 32 and 35 for Ross chickens and on days 1, 7, 14, 21, 28, 32, 35, 42, 49, 56 and 63 for LD chickens.

On sampling day, the live body weight (BW) of each bird was determined to an accuracy of 0.1 g using a mechanical scale (Sartorius, Göttingen, Germany).

The tibiotarsal bones of both left and right legs were excised and cleaned of surrounding muscles and soft tissues and separated from the fibula. Weight (g), relative to body weight (g/100 g BW), length (cm) [between the ends of proximal and distal epiphyses], relative length (cm/100 g BW), mass per unit of length (g/cm) and width (cm) [at the calculated midpoint, i.e. 50% of length] of the left and right tibiotarsal bones were measured and the average values for both bones was calculated. The bones were sealed individually in plastic bags and stored at -20°C until required for further analysis when they were warmed up to 20°C before mechanical testing and microcomputed tomography (μCT) analysis.

### Mechanical properties

**Fig 1:**
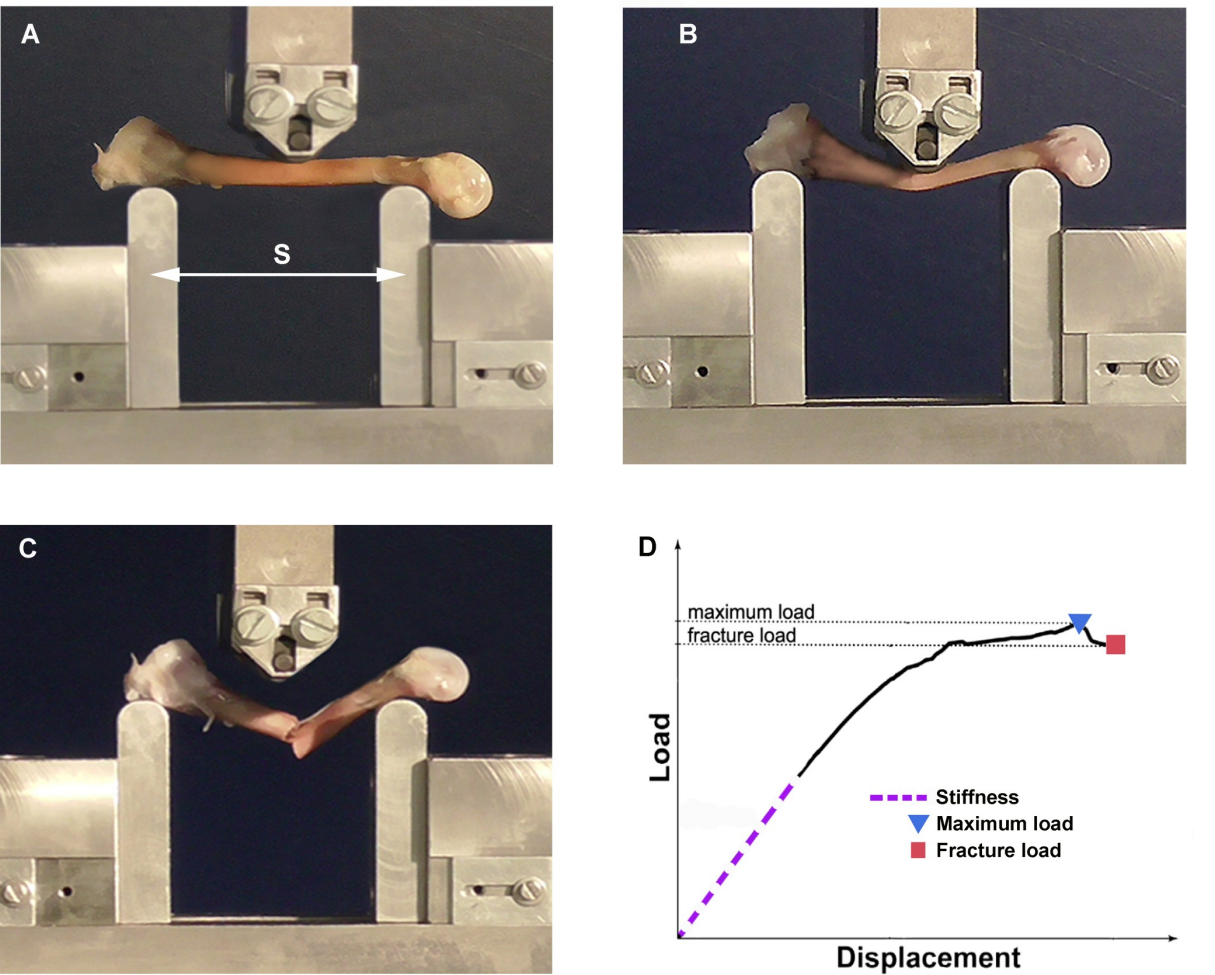
Three-point bending test. (A) Zwick testing machine with the tibiotarsus placed horizontally cranial face down on two support holders and submitted to a vertical force from above. Here (S): span length between two support holders. Zwick testing machine showing (B) maximum load (before the fracture) and (C) fracture load (bone fracture). (D) Load-displacement curve illustrating the estimated mechanical properties.

The mechanical properties of the right tibiotarsal bones were determined by the three-point bending test, using a Zwick testing machine (Zwick/Roell Z010, Ulm, Germany). The cranial face of each tibiotarsus was placed horizontally down on two support holders and submitted to a mid-shaft vertical force (Fig 1A). A span length of 25 mm was used for the tibiotarsi of birds aged from day 1 to day 21 and 50 mm for the bones of older birds. The vertical force testing speed was 0.1 mm.s^-1^ until fracture. The bending force [N] and the displacement (deflection) [mm] were recorded using a TestXpert II software (TestXpert II, Zwick, Ulm, Germany) at a sample rate of 100 Hz. The loading force ranged from 40 N to 1 kN (Fig 1A–1C).

The resulting load-displacement curve was analyzed using a customized Matlab script (The MathWorks, Inc. USA). The following parameters were determined (Fig 1D):

- Stiffness (N/mm): the linear slope of the elastic part of the curve.
- Maximum load (N): the highest load reached.
- Fracture load (N): the load where the bone ultimately failed.

These measurements were normalized for the different span widths according to the equations described previously [32]:

- Rigidity (Nmm^2^) = stiffness (N/mm) × (span length)^3^ / 48
- Maximum bending moment (Nmm) = maximum load (N) × span length / 4
- Fracture bending moment (Nmm) = fracture load (N) × span length / 4

Then these parameters were calculated per unit of BW.

### Microcomputed tomography imaging (μCT)

The μCT analysis was carried out on the left tibiotarsal bones for Ross birds at the ages of 1, 7, 21 and 35 days and for LD birds at the age of 1, 7, 21, 35 and 63 days. The tibiotarsi were scanned using a high-resolution microcomputed tomography (μCT) scanner (VivaCT40, Scanco Medical, Brüttisellen, Switzerland; nominal isotropic image resolution 21 μm, 70 kVp, 114 μA, 381 ms integration time).

**Fig 2:**
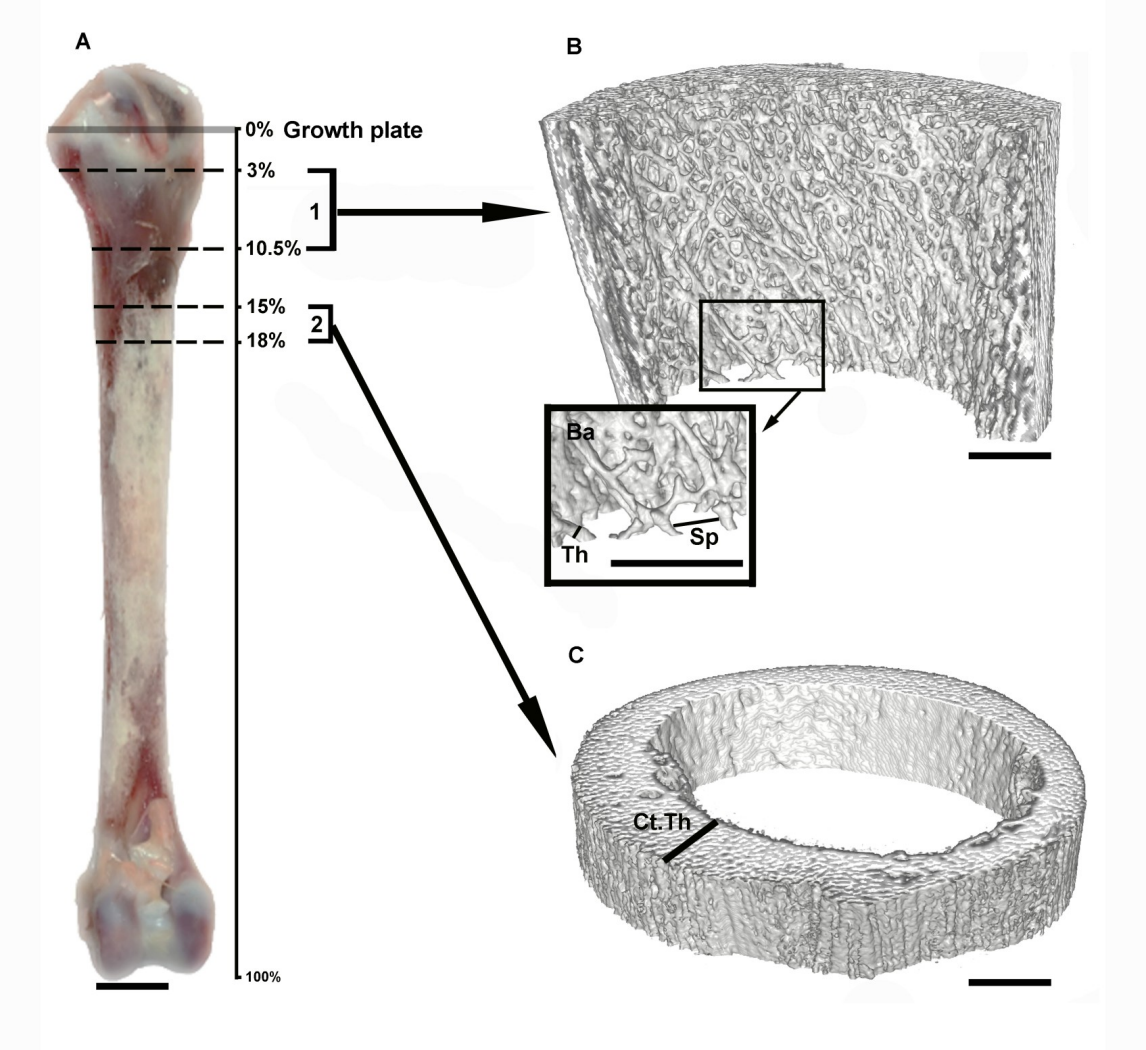
Measurement of the trabecular and cortical bone properties using μCT analysis. (A) Tibiotarsal bone of LD chicken at day 63: (1) the trabecular sample taken from 3% below the distal-most point of the growth plate and extended distally for 7.5% of the tibiotarsus length. (2) The cortical region of interest selected from the diaphyseal region from 15% distal to the proximal growth plate and extending distally 3% of the tibiotarsus length. (B and Ba) Trabecular bone. (C) Cortical bone. Ct.Th: cortical thickness, Th: trabecular thickness and Sp: trabecular separation. Bar = 1 cm for A, and 1 mm for B, Ba and C.

Microcomputed tomography analysis was performed on trabecular and cortical regions of interest selected using ImageJ software (ImageJ 1.52h, Wayne Riband, National Institutes of Health, USA) according to the methodologies reported by Castrillón et al [33]. Trabecular bone samples [trabecular ROI(region of interest)] were taken from 3% below the distal-most point of the proximal growth plate and extended distally for 7.5% of the tibiotarsus length. Cortical bone samples (cortical ROI) were obtained from the diaphyseal region from 15% distal to the proximal growth plate and extended distally 3% of the tibiotarsus length [33] (Fig 2A–2C).

To segment the bone from marrow and soft tissue, firstly the colour threshold was measured manually for each bird using the gray scale. Thereafter, the average of the colour threshold values of each individual age group were calculated. Then, the software generated the estimated parameters based on the previously calculated threshold.

The following parameters were measured for the trabecular ROI:

- Volumetric bone mineral density (Tb.BMDv; mg/cm^3^): Mass of mineralized bone per total volume in the trabecular ROI.
- Bone volume fraction (BV/TV; %): Ratio of the segmented bone volume to the total volume of the trabecular ROI.
- Trabecular thickness (Tb.Th; mm) (Fig 2Ba).
- Trabecular number (Tb.N; 1/mm).
- Trabecular separation (Tb.Sp; mm) (Fig 2Ba).

Trabecular bone analysis was performed only from day 7 onward due to the scarcity of trabeculae on day 1, which could have led to unreliable results [28].

For the cortical ROI, the parameters measured included:

- Volumetric bone mineral density (Ct.BMDv; mg/cm^3^): Mass of mineralized bone per total volume in the cortical ROI.
- Cortical thickness (Ct.Th; mm) (Fig 2C).
- Cortical bone area (Ct.Ar; mm^2^).
- Total cross-sectional area (Tt.Ar; mm^2^).
- Cortical area fraction (Ct.Ar/Tt.Ar; %).
- Medullary section area (Med.Ar; mm^2^) was calculated as follows:

Med.Ar = Tt.Ar—Ct.Ar

### Statistical analysis

Statistical analyses were performed using the statistical package program IBM SPSS Statistics 23 (IBM Corporation, New York, USA). The graphs were generated using the statistical package program JMP® Pro 13 (SAS Institute Inc., Cary, USA). Comparison of the data between the two lines at the same age were performed using the Mann–Whitney U test. One-way analysis of variance (ANOVA) with the post hoc Dunnett’s test was performed to evaluate the effect of age on the tibiotarsus parameters. Pearson's correlation coefficient was used to test the relation between mechanical properties (rigidity, M-Max and M-fracture) and the other densitometric and geometric parameters as well as between Tb.BMDv and Ct.BMDv. To explore the effect of chicken line and BW on the tibiotarsus parameters, all data collected was regressed against the chicken line and the BW using the log-log regression model. All statistical analyses were two-sided with significance defined as a p-value of < 0.05.

## Results

### Morphometric properties

**Fig 3:**
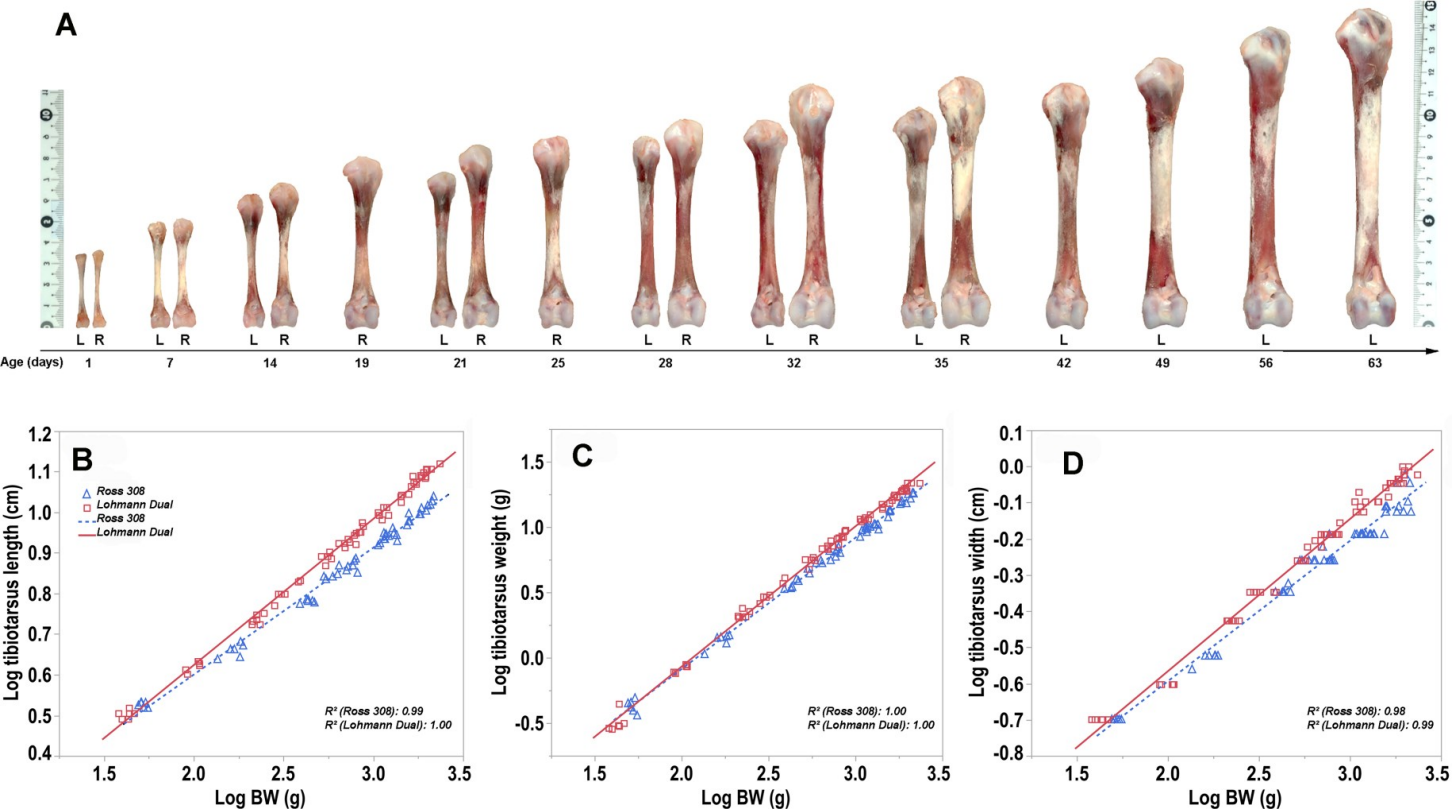
(A) Tibiotarsus length of Ross (R) and LD (L) chicken lines. (B-D) Allometric plots: log transformed length, weight and width of the tiobiotarsus versus log of body weight (BW) post hatching for Ross and LD chicken lines. Symbols represent each individual value for each chicken line.

Length (Fig 3A), width and weight of the tibiotarsus increased steadily with age in both lines (p < 0.05). From day (d) 1 to d 35 post hatching, the length and the weight of the tibiotarsus of Ross chickens increased at a rate of 0.21 cm/d and 0.49 g/d, respectively, whereas the LD chickens tibiotarsus increased by a rate of 0.17 cm/d and 0.23 g/d over the same period, and from d 1 to 63 the increase was by a rate of 0.15 cm/d and 0.32 g/d. For Ross birds, the highest rate of increase in the bone length (0.23 cm/d) and weight (1 g/d) was between d 28–35, while for LD birds, the highest rate of increase in the bone length (0.2 cm/d) was between d 21–28 and the greatest increase in bone weight (0.52 g/d) was between d 42–49.

The tibiotarsus of LD chickens was significantly shorter, thinner and lighter than those of Ross chickens at all ages between d 1and d 35 post hatching (Table 1). However, when length and weight of the tibiotarsus were expressed relative to body weight, the tibiotarsus of LD chickens had a greater relative length, width and weight than Ross chickens at all age groups (p < 0.05) (Table 1).

**Table 1:** Body weight, tibiotarsus length, weight and width of LD and Ross chicken lines versus day post hatching.

Age (day)	Line (n)	BW (g)		Tibiotarsus length (cm)		Tibiotarsus weight (g)		Tibiotarsus width (cm)	
		Means	SD	Means	SD	Means	SD	Means	SD
**1**	**Ross (6)**	52.26	2.29	3.36	0.05	0.43	0.04	0.20	0.01
	**LD (6)**	42.45	3.06	3.18	0.08	0.32	0.01	0.20	0.01
**7**	**Ross (6)**	169.47	19.41	4.58	0.17	1.36	0.12	0.30	0.01
	**LD (6)**	101.20	8.12	4.19	0.12	0.84	0.06	0.25	0.01
**14**	**Ross (6)**	435.35	28.03	6.04	0.06	3.61	0.20	0.45	0.01
	**LD (6)**	224.77	13.01	5.45	0.15	2.15	0.12	0.38	0.01
**19**	**Ross (6)**	640.73	110.77	7.04	0.17	5.34	0.57	0.55	0.01
**21**	**Ross (6)**	746.58	58.32	7.45	0.18	6.35	0.43	0.58	0.04
	**LD (6)**	329.17	46.05	6.39	0.34	3.22	0.41	0.45	0.01
**25**	**Ross (6)**	1191.67	92.54	8.60	0.14	9.63	0.25	0.65	0.01
**28**	**Ross (6)**	1221.00	108.99	8.82	0.28	10.00	0.56	0.65	0.01
	**LD (6)**	575.33	62.55	7.82	0.27	5.75	0.46	0.57	0.03
**32**	**Ross (6)**	1677.83	172.74	9.71	0.32	13.71	0.70	0.74	0.05
	**LD (6)**	754.17	93.82	8.59	0.41	7.71	0.64	0.65	0.03
**35**	**Ross (6)**	2013.17	142.70	10.43	0.36	17.02	0.87	0.85	0.08
	**LD (6)**	791.67	58.41	8.86	0.35	8.21	0.41	0.65	0.01
**42**	**LD (6)**	1130.50	59.16	9.98	0.28	11.66	0.27	0.79	0.04
**49**	**LD (6)**	1522.50	112.73	11.19	0.46	15.33	1.22	0.83	0.04
**56**	**LD (6)**	1817.33	134.21	12.20	0.35	18.60	1.47	0.92	0.03
**63**	**LD (6)**	2011.83	182.87	12.64	0.37	20.16	1.06	0.95	0.04

BW: live body weight; LD: Lohmann Dual; Line: genetic line; n: animal number; Ross: Ross 308; SD: standard deviation of the mean.

**Fig 4:**
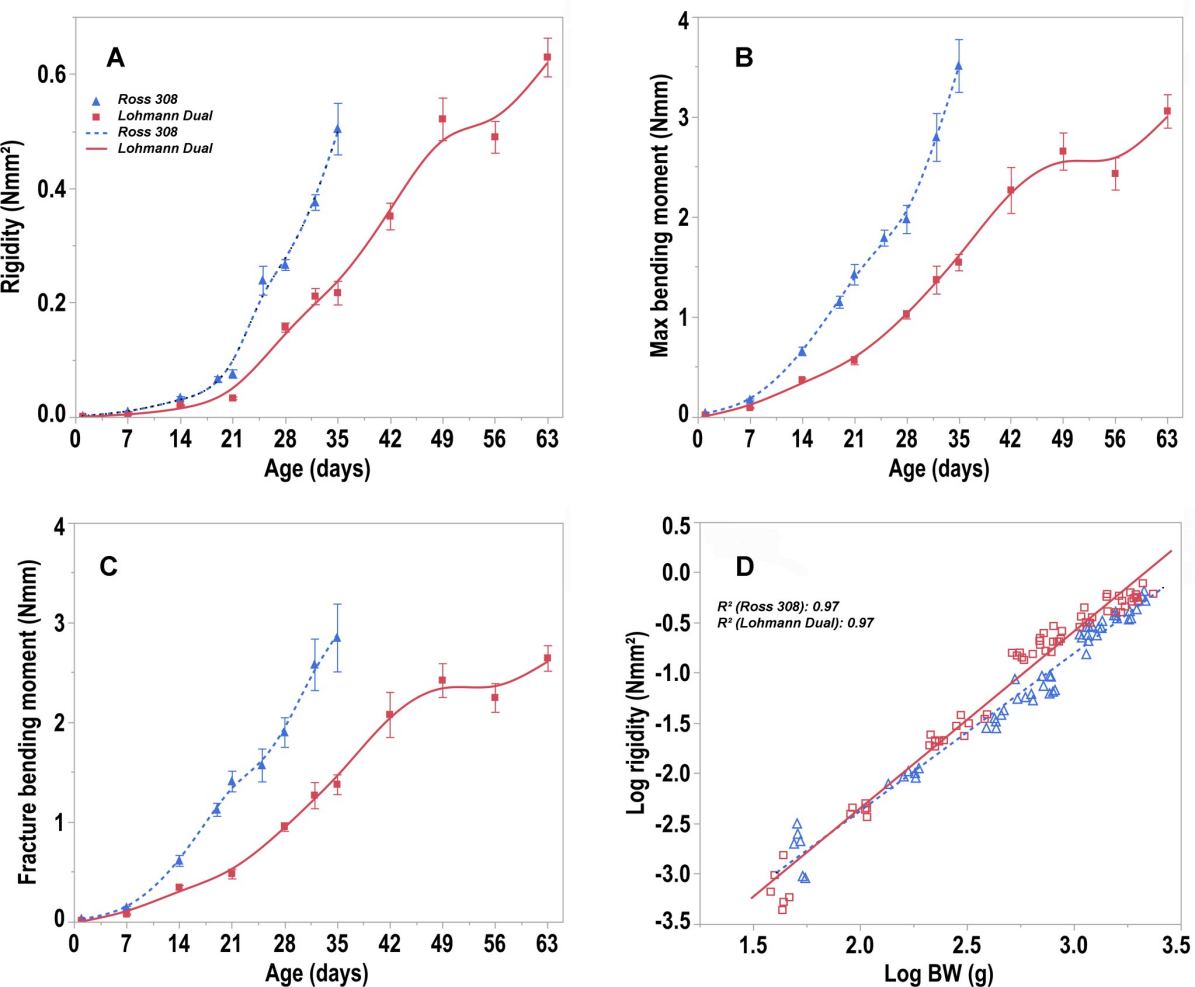
Trendlines of the changes in the mechanical properties of the tibiotarsus versus day post hatching. (A) rigidity, (B) maximum bending moment and (C) fracture bending moment of the tibiotarsus versus day post hatching for Ross and LD chicken lines. Bars refer to mean ± standard error of the mean of the chicken samples at each time interval. (D) allometric plot: log transformed rigidity versus log of body weight (BW) post hatching for Ross and LD chicken lines. Symbols represent each individual value for each chicken line.

The relative tibiotarsus length of both chicken lines decreased significantly over the entire study period for Ross chickens and until day 42 for LD chickens, thereafter the decrease was not significant. The relative tibiotarsus width of both chicken lines decreased until day 28 for both chicken lines, subsequently the relative tibiotarsus width did not change significantly. The relative tibiotarsus weight of both chicken lines did not differ over the study period except between d 7 and d 14 for LD chickens (Fig 4).

Regression analysis showed that the chicken line had an effect on the length, weight and width of the tibiotarsus, where the tibiotarsus of LD chickens had a greater length by 6%, weight by 7% and width by 5.2% than those of Ross chickens of the same BW (p < 0.001), adjusted R^2^ = 0.99, 0.99 and 0.98, respectively (Fig 3B–3D).

The tibiotarsus mass per unit of length in Ross chickens increased with age over the whole study period and in LD chickens until d 56 (p < 0.05). The tibiotarsus mass per unit of length of LD chickens was significantly lower than that of Ross chickens in all comparable age groups between d 1 and d 35 post hatching (p < 0.05).

### Mechanical properties

Rigidity, M-Max and M-fracture of the tibiotarsus all increased with age. The LD chickens' tibiotarsus had a lower: rigidity, M-Max and M-fracture than those of Ross chickens in all age groups (Fig 4A-4C) (p < 0.05). However, there were no differences between both chicken lines, when these parameters were expressed relative to body weight.

Furthermore, at the same BW, the chicken line had an influence on the tibiotarsus rigidity (p < 0.001). The tibiotarsus rigidity of LD chickens was greater on average by 1.7% than that of Ross chickens, R^2^ = 0.97 (Fig 4D). The correlations between the mechanical properties and the other densitometric and geometric parameters are presented in Table 2.

**Table 2:** Pearson’s correlation coefficient (r) between the mechanical properties and the other densitometric and geometric parameters of LD and Ross chicken lines.

Parameters	Line	BMDv trab	BMDv cortical	Ct.Th (mm)	B.Ar (mm^2^)	Med.Ar (mm^2^)	Tib.Den (g/cm)	Tib.Lth (cm)	Tib.Wth (cm)	Tib.Wt (g)	BW (g)
**BW (g)**	**Ross**	0.61	0.84	0.87	0.94	0.94	0.99	0.98	0.97	0.99	1
	**LD**	0.95	0.88	0.92	0.96	0.94	0.99	0.99	0.99	0.99	1
**Rigidity (Nmm^2^)**	**Ross**	0.88	0.59	0.64	0.90	0.94	0.92	0.94	0.86	0.96	0.96
	**LD**	0.81	0.66	0.82	0.94	0.91	0.95	0.97	0.93	0.97	0.97
**M-max (Nmm)**	**Ross**	0.85	0.66	0.74	0.97	0.99	0.96	0.98	0.97	0.97	0.96
	**LD**	0.89	0.73	0.89	0.98	0.91	0.96	0.95	0.95	0.94	0.95
**M-fracture (Nmm)**	**Ross**	0.78	0.65	0.71	0.95	0.95	0.93	0.94	0.91	0.92	0.92
	**LD**	0.89	0.73	0.90	0.98	0.90	0.96	0.95	0.95	0.94	0.94

All correlations were significant at p-value ≤0.01.

B.Ar: cortical bone area; BMDv cortical: volumetric bone mineral density of cortical bone; BMDv trab: volumetric bone mineral density of trabecular bone; BW: body weight; Ct.Th: cortical thickness; LD: Lohmann Dual; Line: genetic line; Med.Ar: medullary section area; M-fracture: fracture bending moment; M-max: maximum bending moment; Ross: Ross 308; Tib.Den: weight (g) per 1 cm of the tibiotarsus; Tib.Lth: tibiotarsus length; Tib.Wth: tibiotarsus width; Tib.Wt: tibiotarsus weight.

### Trabecular bone structural properties

The volumetric bone mineral density (BMDv) increased steadily with age over the entire investigation in the LD birds (p < 0.05). However, in Ross birds the tibiotarsus did not change from d 1 to d 21, but increased from d 21 to d 35 (p < 0.05). Following day one, there were no differences in the volumetric bone mineral density of trabecular bone between the two lines (Table 3). According to the regression analysis, the chicken line had an influence on the trabecular BMDv (p < 0.001). The trabecular BMDv of LD chickens was greater, on average, by 3.2% than that of Ross chickens of the same BW, R^2^ = 0.74.

**Table 3:** Volumetric bone mineral density of trabecular and cortical bone of LD and Ross chicken lines versus day post hatching.

Age (d)	Line (n)	BMDv trabecular (g/cm^3^)		BMDv cortical (g/cm^3^)	
		Mean	SD	Mean	SD
**1**	**Ross (6)**	433.60	23.43	522.59	61.75
	**LD (6)**	350.10	34.08	535.68	35.18
**7**	**Ross (6)**	422.59	17.71	685.27	8.47
	**LD (6)**	431.56	21.60	718.55	31.51
**21**	**Ross (6)**	443.93	41.04	692.41	38.09
	**LD (6)**	497.87	20.83	703.31	13.21
**35**	**Ross (6)**	573.79	29.25	757.72	36.47
	**LD (6)**	589.61	31.79	781.33	30.00
**63**	**LD (6)**	647.02	18.94	811.61	11.57

BMDv cortical: volumetric bone mineral density of cortical bone; BMDv trabecular: volumetric bone mineral density of trabecular bone; LD: Lohmann Dual; Line: genetic line; n: animal number; Ross: Ross 308; SD: standard deviation of the mean.

The bone volume fraction of the LD tibiotarsus decreased between d 7 and d 21 post hatching (p < 0.05). Thereafter, it remained unchanged till the end of the study. From d 7 to d 35, the bone volume fraction of the Ross tibiotarsus decreased (p < 0.05). Excluding d 21, no differences in the bone volume fraction of the tibiotarsus between the LD and Ross chickens were observed over the study period (Table 4).

**Table 4:** Trabecular bone properties of LD and Ross chicken lines at different ages.

Age (d)	Line (n)	BV/TV (%)		Tb.Nb (1/mm)		Tb.Th (mm)		Tb.Sp. (mm)	
		Mean	SD	Mean	SD	Mean	SD	Mean	SD
**1**	**Ross (6)**	26.55	4.47	1.41	0.35	0.15	0.02	0.59	0.15
	**LD (6)**	28.75	2.11	2.40	0.14	0.15	0.03	0.27	0.03
**7**	**Ross (6)**	48.64	6.10	3.35	0.49	0.12	0.01	0.18	0.05
	**LD (6)**	45.44	12.24	2.93	0.81	0.13	0.03	0.25	0.15
**21**	**Ross (6)**	41.47	5.28	1.70	0.57	0.12	0.01	0.54	0.26
	**LD (6)**	36.13	2.31	1.26	0.16	0.12	0.01	0.69	0.10
**35**	**Ross (6)**	28.32	2.16	0.89	0.23	0.13	0.01	1.08	0.37
	**LD (6)**	30.04	3.95	0.76	0.24	0.13	0.01	1.30	0.44
**63**	**LD (6)**	29.97	2.52	0.70	0.18	0.15	0.01	1.34	0.36

BV/TV: bone volume fraction; LD: Lohmann Dual; Line: genetic line; n: animal number; Ross: Ross 308; SD: standard deviation of the mean; Tb.Sp.: trabecular separation; Tb.Nb: trabecular number; Tb.Th: trabecular thickness.

Trabecular number of the tibiotarsus in both chicken lines decreased from d 7 to d 35 (p < 0.05). Between d 35 and d 63, the values did not change in the LD tibiotarsus. Trabecular thickness of the tibiotarsus in both lines did not alter from d 7 post hatching until the end of the study. Trabecular separation in the LD tibiotarsus increased from d 7 to d 35 (p < 0.05). Between d 35 and d 63, the values did not differ. For the Ross tibiotarsus, trabecular separation increased from d 7 to d 35 (p < 0.05). There were no differences in trabecular number, trabecular thicknesses and trabecular separation between LD and Ross chickens over the whole study period (p > 0.05) (Table 4).

### Cortical bone structural properties

The cortical BMDv in both lines increased from d 1 to d 7 (p < 0.05), then did not differ between d 7 and d 21. From d 21 to d 35, it increased again (p < 0.05). For the LD tibiotarsus, the values did not change from d 35 to d 63. No line differences in the volumetric bone mineral density of cortical bone were found in any age groups (Table 3). According to the regression analysis, the chicken line had an influence on the cortical BMDv (p < 0.001). The cortical BMDv of LD chickens was greater, on average, by 3.5% than Ross chickens of the same BW, R^2^ = 0.71. The correlation between the trabecular BMDv and the cortical BMDv of the tibiotarsus was positive in both chicken lines and greater in LD chickens than in Ross chickens, r = 0.91 and 0.66 for LD and Ross lines, respectively.

The cortical thickness of the tibiotarsus in both chicken lines increased from d 1 to d 35 (p < 0.05), then it remained unchanged until d 63 for LD chickens. There were no differences in cortical thickness between LD and Ross chicken lines at the same age over the study (Table 5).

**Table 5:** Cortical bone properties of LD and Ross chicken lines at different ages.

Age (d)	Line (n)	Ct.Th (mm)		B.Ar (mm^2^)		T.Ar (mm^2^)		Ct.fraction (%)		Med.Ar (mm^2^)	
		Mean	SD	Mean	SD	Mean	SD	Mean	SD	Mean	SD
**1**	**Ross (6)**	0.20	0.02	1.68	0.14	5.24	0.57	0.32	0.03	3.56	0.52
	**LD (6)**	0.19	0.01	1.37	0.10	5.25	0.49	0.26	0.02	3.88	0.46
**7**	**Ross (6)**	0.29	0.04	4.04	0.57	12.54	1.26	0.32	0.04	8.5	1.06
	**LD (6)**	0.24	0.02	2.66	0.25	9.86	0.51	0.27	0.03	7.2	0.53
**21**	**Ross (6)**	0.34	0.05	14.97	1.75	44.25	3.88	0.34	0.02	29.3	2.42
	**LD (6)**	0.29	0.03	9.82	1.16	29.04	3.45	0.34	0.03	19.2	2.77
**35**	**Ross (6)**	0.38	0.04	26.29	1.06	86.53	7.84	0.31	0.02	60.24	7.02
	**LD (6)**	0.41	0.04	15.91	0.91	53.24	4.56	0.30	0.03	37.34	4.69
**63**	**LD (6)**	0.45	0.05	27.45	2.25	90.03	17.85	0.32	0.10	62.6	17.93

B.Ar: cortical bone area; Ct.fraction: cortical area fraction; Ct.Th: cortical thickness; LD: Lohmann Dual; Line: genetic line; Med.Ar: medullary section area; n: animal number; Ross: Ross 308; SD: standard deviation of the mean; T.Ar: total cross-section area.

The cortical bone area, total cross-sectional area and medullary section area of both LD and Ross chicken lines increased with age over the study period (p < 0.05). The LD tibiotarsus had a lower cortical bone area at all ages, a lower total cross-sectional area on days 21 and 35 and a lower medullary section area from d 7 onwards than those of the Ross tibiotarsus (p < 0.05) (Table 5). Between d 1 and d 7 the cortical area fraction of the LD tibiotarsus remained unchanged, thereafter it increased gradually until d 21. From d 21 onwards the cortical area fraction of the LD tibiotarsus did not change. The cortical area fraction of the Ross tibiotarsus remained unchanged over the study period. On days 1 and 7, the LD tibiotarsus had a lower cortical area fraction than those of the Ross tibiotarsus (p < 0.05). Thereafter, there were no differences in the cortical area fraction of the tibiotarsus between both chicken lines (Table 5).

## Discussion

The choice to examine the tibiotarsal bone in this study was based on earlier research findings in poultry, that birds genetically selected for rapid growth and heavy muscle mass have tibiotarsi that are greatly stressed and are prone to mineralization disorders and fractures [10, 14, 34]. During growth, the skeleton of fast-growing chickens must adapt and modify its morphology and material properties to successfully withstand the effects of their rapidly increasing body weight [35].

These observations validate the results of the present investigation, where the tibiotarsal; length, width and weight of both Ross and LD chicken lines similar strongly correlated with the BW (Table 2), indicating that the growth of the tibiotarsus had a similar pattern of growth in both genetic lines. When both LD and Ross chicken lines had the same age, the tibiotarsus of Ross chickens was longer, thicker and heavier than that of LD chickens. However, when length, width and weight of the tibiotarsus were expressed relative to body weight, the tibiotarsus of Ross chickens was shorter, thinner and lighter than that of LD chickens. This could be due to the metabolic inability to support optimal growth in tibiotarsus length, width and weight at the same rate as muscle growth in fast-growing chickens [19]. In this study the LD chicken line had longer, thicker and heavier tibiotarsi than those of the Ross chicken line at the same BW, as reported previously in findings between unselected and selected chickens for meat production [25].

The increase in the tibiotarsal: cortical thickness, total bone cross section area, cortical area fraction and medullary section area over time followed a similar pattern in both chicken lines. The increase in the total bone cross section area is caused by, in part, periosteal apposition with the production of new osteons at the periosteal surface, whereas the increases in the medullary section area are due to endosteal resorption through increased osteoclastic activity at the endosteal surface [31].

The cortical area fraction remained constant with age in both LD and Ross chicken lines, indicating that total bone and medullary cross section areas in both chicken lines correlated strongly positive with each other. However, total bone width increased more rapidly in Ross chickens than in LD chickens, which is in agreement with previous studies that compared fast with slow-growing chickens [13, 19, 36]. Although the bone width was greater in Ross chickens than in LD chickens of the same age, the cortical thickness was similar in both chicken lines over the study period. This indicates that the medullary section area in Ross chickens is greater than in LD chickens. LeBlanc et al. (1985) who compared fast- and slow-growing turkey genotypes also found that the cortical thickness of the tibiotarsus was similar in both genotypes [8]. In contrast, William et al. (2000) used two distinct genetic lines of Ross birds; a slow-growing chicken line not selected for growth performance since 1972 and a modern fast-growing chicken line selected for rapid growth, efficient food conversion and optimal skeletal quality [19]. They found that fast-growing chickens had a thicker cortical thickness than did slow-growing chickens. They concluded that the greater cortical thickness is an essential element for the optimal dimensions of the tibiotarsus to support the rapid increase in body weight in fast-growing chickens [19].

The proximal metaphysis is preferred for μCT analysis of avian trabecular bone because it contains a large amount of trabecular bone that distributes impact loads applied to the cortex thus contributing appreciably to the mechanical strength of the long bones [37–39]. Trabecular bone analysis was performed only from day 7 onward due to the scarcity of trabeculae on day 1. Yair et al. (2013) were also not able to analyze the trabecular properties in chickens before day 7 of age [28]. They attributed that to the impaired bone development during the perinatal period. It has been supposed that one of the reasons of this “slow-down” phenomenon is the nutrient depletion seen prenatally leading to impaired bone development [40].

The results of this study showed that the tibiotarsus of both chicken lines had a reduction in trabecular numbers over time with unchanged trabecular thickness. Consequently, the trabecular separation increased resulting in a decreased bone volume fraction with age. Similar growth patterns of the trabecular bone have been reported recently in chickens [28], geese [41] and in humans [42]. The tibiotarsal mechanical properties such as rigidity, maximal strength, and fracture strength are indicators of skeletal integrity and associated with differing bone characteristics including both densitometric (cortical and trabecular BMDv) and geometric parameters (bone weight, bone weight per unit of length, bone width, total and cortical bone area) [23, 32, 43]. Our results show that the bone volume fraction decreases with age in both chicken lines, resulted from the reduction in trabecular numbers over time with unchanged trabecular thickness, which could diminish the bone fracture strength. However, cortical and trabecular BMDv inversely increased thus enhancing the bone strength.

Williams et al. (2004) investigated the tibiotarsus growth in a fast-growing chicken line selected for optimal weight gain and skeletal quality. They found that although the morphological properties of the tibiotarsus in the fast-growing chickens correlate with the rapid weight increase, the tibiotarsus of the fast-growing chickens was less mineralized than that of the slow-growing chickens [13]. Consequently, they hypothesized that the tibiotarsus of the fast-growing chickens would have a lower bone fracture strength. Contrary to this, McDevitt et al. (2006) found that the bone fracture strength of the tibiotarsus of fast-growing chickens was higher than that of slow-growing chickens at the same age [25]. They explained that the tibiotarsus of fast-growing chickens was heavier and had greater bone mineral density than did the tibiotarsus of slow-growing chickens at the same age. McDevitt et al. (2006) measured the actual bone fracture strength using the three-point bending test, whereas Williams et al. (2004) suggested indirectly that the bone would have a lower effective bone fracture strength. We support the conclusion of McDevitt et al. (2006) because the tibiotarsal bone strength is influenced by many factors including bone weight, structure, and composition [25–27]. In the present study, the tibiotarsi of the Ross chickens were twice as strong as those of the LD chickens at the same age. Here the tibiotarsi of Ross chickens had a greater mass per unit of length, greater width and greater cortical cross section area than those of LD chickens at the same age.

Shim et al. (2012) and Rawlinson et al. (2009) reported a negative correlation between tibiotarsal fracture strength and growth rate, where slow-growing chickens had a greater relative bone fracture strength than that of fast-growing chicken [20, 36]. They showed that bone mineral density correlated negatively with growth, i.e. in age-matched birds the fast-growing chickens had a relatively lower bone mineral density than that of the slow-growing chickens. In contrast, our results showed a positive correlation between tibiotarsal fracture strength and growth rate in both chicken lines resulting from the similar relative mechanical properties of both chicken lines. Furthermore, the cortical and trabecular BMDv and the cortical thickness were similar in both chicken lines at the same ag over the study.

This study demonstrated that the tibiotarsal bone of the novel dual-purpose chicken line, LD, had a similar growth pattern to that of the Ross broiler chicken line. Furthermore, at the same BW, the tibiotarsus of LD chickens had a greater rigidity than that of Ross chickens. We suggest that this is due to the superior morphometric properties (weight, width and length) and microarchitecture parameters (cortical and trabecular bone BMDv) of the LD chickens when compared to those of the Ross chickens at the same body weight. These conclusions support the finding that the tibiotarsal bone of the LD chicken line had more bending resistance than did that of Ross chickens. Consequently, growing LD chickens to a similar BW to that of Ross chickens at the time of normal commercial slaughter will not affect their leg skeleton stability.

## List of abbreviations

BV/TV: bone volume fraction
BW: body weight
Ct.Ar: Cortical bone area
Ct.Ar/Tt.Ar: Cortical area fraction
Ct.BMDv: Volumetric bone mineral density in the cortical bone
Ct.Th: Cortical thickness
d: day
LD: Lohmann Dual
M-fracture: fracture bending moment
M-Max: maximum bending moment
Med.Ar: Medullary section area
N: newton
ROI: region of interest
Ross: Ross 308
Tb.BMDv: Volumetric bone mineral density in the trabecular bone
Tb.N: Trabecular number
Tb.Sp: Trabecular separation
Tb.Th: Trabecular thickness
Tt.Ar: Total cross-sectional area
μCT: microcomputed tomography
